# ‘Is totally endoscopic coronary artery bypass grafting compared with minimally invasive direct coronary artery bypass grafting associated with superior outcomes in patients with isolated left anterior descending disease?’

**DOI:** 10.1016/j.amsu.2020.07.060

**Published:** 2020-08-11

**Authors:** Lucy Manuel, Laura S. Fong, Hugh Wolfenden, Levi Bassin

**Affiliations:** a Department of Cardiothoracic Surgery, Royal North Shore Hospital, Sydney, Australia; b Department of Cardiothoracic Surgery, Prince of Wales Hospital, Sydney, Australia

**Keywords:** Review, Coronary artery bypass, Robotics, MIDCAB, TECAB

## Abstract

A best evidence topic in cardiac surgery was written according to a structured protocol. The question addressed was ‘Is totally endoscopic coronary artery bypass grafting compared with minimally invasive direct coronary artery bypass grafting associated with superior outcomes in patients with isolated left anterior descending disease?’ Altogether more than 118 papers were found using the reported search, of which 4 represented the best evidence to answer the clinical question, which included 2 prospective cohort studies and 2 retrospective observational studies. The authors, journal, date and country of publication, patient group studied, study type, relevant outcomes and results of these papers were tabulated. There is a significant variation within the MIDCAB and TECAB techniques amongst the studies-including the experience of the surgeon, use of cardiopulmonary bypass, patient selection, and target vessel grafting strategies-highlighting the complexity of comparing these two minimally invasive procedures. Operative times were comparable across all studies, with TECAB patients having higher transfusions rates and conversion rates to either a median sternotomy or MIDCAB procedure. Overall safety was comparable between the two cohort groups, with similar length of stay and 30-day mortality. However, the TECAB group were more likely to require re-operation for bleeding and reintervention for early revascularisation with greater total hospital costs than the MIDCAB patients. Based on the available evidence, we conclude that TECAB is associated with a higher rate of transfusions, conversion to median sternotomy or MIDCAB, early graft failure and reintervention compared to the MIDCAB approach. We advise caution in adopting a TECAB approach.

## Highlights

• Coronary Artery Bypass Grafting remains the gold standard for complex multi-vessel disease.
• Demand for minimally invasive and robotic procedures is increasing.
• Controversy remains regarding the optimal method for robotically assisted coronary grafting.
• Overall safety is comparable between TECAB and MIDCAB.
• TECAB is associated with a higher rate of early graft failure and reintervention.

## 1 Introduction

A best evidence topic was constructed according to a structured protocol. This is fully described in a previous publication in the IJS [1].

## 2 Clinical scenario

A 53-year-old male requires coronary artery revascularisation for unstable angina with a 95% proximal left anterior descending (LAD) artery stenosis. He requests a minimally invasive robotic approach in order to hasten his return to work and post-operative recovery. You plan to offer your patient a MIDCAB procedure, however, your colleague has had excellent results with a TECAB approach. You resolve to review the literature to determine if one approach is superior.

## 3 Three-part question

In patients with isolated left anterior descending disease is totally endoscopic coronary artery bypass grafting (TECAB) superior to minimally invasive direct coronary artery bypass grafting (MIDCAB) in terms of peri-operative outcomes including requirement for reintervention/target vessel revascularisation, freedom from major adverse cardiac and cerebrovascular events, and survival?

## 4 Search strategy

A literature search was performed on the MEDLINE database (1964 to present) using the OVID interface with the terms ‘coronary artery bypass’ [all fields] OR ‘coronary’ [all fields] OR ‘MIDCAB’ [all fields] OR ‘TECAB’ [all fields] AND ‘robotics or robotic surgical procedure’ [all fields]. The reference lists of initially identified papers were searched for other relevant studies. The search was current as of June 23, 2020.

## 5 Search outcome

A total of 118 papers were found using the reported search. From these 5 were not in English, 69 were irrelevant, 12 were case reports or editorial commentary, 15 focused on hybrid robotic procedures utilising PCI, and 13 were not specific to either procedure or failed to compare the two groups of interest. The remaining 4 papers directly compared TECAB and MICAB procedures and were therefore chosen as the best evidence to answer the clinical question.

## 6 Results

The results of the four papers (two prospective cohort studies and two retrospective observational studies) are summarised in Table 1.

**Table 1:** Best evidence papers.

Author, date, journal and country, study type (level of evidence)	Patient group	Outcomes	Key results TECAB vs. MIDCAB	Comments
Kofler et al., 2017, Innovations Austria [2] Prospective cohort study (level II)	264 patients with single or sequential IMA grafts to anterior wall TECAB: 204 MIDCAB: 60 Mean follow-up: 36 months	Mean operative time (min)	292 vs. 201 **(p < 0.001)**	This study demonstrated significantly longer operative times in the TECAB group. However, there was no significant difference in survival and freedom from MACCE at 36 months between the two groups. Patients unsuitable for remote access perfusion were assigned to MIDCAB, leading to a potential bias towards TECAB with relatively higher risk patients (older age, diabetes mellitus, renal insufficiency, dialysis, peripheral vascular disease, poor LV function) being assigned to the MIDCAB procedure
		Mean CPB time (min)	93 vs. 39 (p = 0.045)	
		AXC time (min)	56 vs. 12 (p = 0.011)	
		Conversion rates %	18% vs. 3% **(p < 0.001)**	
		RBC Transfusion	42% vs. 10% **(p < 0.001)**	
		FFP Transfusion	22% vs. 3% **(p < 0.001)**	
		PLT Transfusion	3% vs. 7% (p = 0.114)	
		MI	1.5% vs. 0 (p = 0.463)	
		Stroke	1.5% vs. 0 (p = 0.454)	
		Hospital length of stay (days)	7 vs. 6 (p = 0.716)	
		Mortality (36 months)	1.5% vs. 1.7% (p = 0.298)	
		Freedom from major adverse cardiac and cerebrovascular events (36 months)	12.4% vs. 5.1% (p = 0.358)	
		Target Vessel Revascularisation (36 months)	1% vs. 3.4% (p = 0.114)	
Yang et. Al, 2015, The Annals of Thoracic Surgery, China [3] Prospective cohort study (level II)	240 patients with single vessel LAD or proximal RCA disease, and multi-vessel disease in which LAD was involved while non-LAD disease was amendable to PCI TECAB: 100 MIDCAB: 140 Mean follow-up: 41.1 months	Operative times (min)	219 vs. 264 **(p < 0.001)**	This study demonstrated significantly longer operative times with the MIDCAB group. No comparative statistical analysis of data was performed for outcomes
		Re-operation for bleeding	1% vs. 0	
		Post-operative infection	0 vs. 2.9%	
		Staged PCI	10% vs. 7.9%	
		IMA patency (3 years)	97.1% vs 96.4%	
		Target Vessel Revascularisation (3 years)	2% vs. 0.71%	
Pasrija et. Al, 2018, Innovations, USA [4] Retrospective observational study (level III)	100 patients undergoing robotically assisted single LIMA- LAD revascularisation TECAB: 50 MIDCAB: 50	Operative time (hr)	3.5 vs. 3.3 (p = NS)	Despite the MIDCAB group containing less experienced surgeons (<75 cases vs. TECAB >200 cases), there was no significant difference in in-hospital mortality between the two groups.
		CPB use	56% vs. 0 **(p < 0.001)**	
		Conversion rates %	4% vs. 0 (p = NS)	
		Ventilator time (hr)	8.5 vs. 4 **(p < 0.001)**	
		Total blood products	2 vs. 0 **(p < 0.001)**	
		30-day/in-hospital mortality	2% vs. 0 (p = NS)	
		Reintervention	2% vs. 2% (p = NS)	
		Total cost ($US)	33 769 vs. 22 679 **(p < 0.001)**	
Jegaden et. Al, 2011, Journal of Cardiothoracic Surgery, France [5] Retrospective observational study (level III)	160 patients undergoing elective LAD minimally invasive revascularisation TECAB: 59 MIDCAB: 53 PA-CABG: 48	Operative times (hr)	3.4 vs. 3.1 (p = NS)	This study demonstrated no significant difference between operative times and reoperation for bleeding between the three groups. However, there was a significantly higher rate of reintervention in the TECAB group.Unfortunately, this study included PACAB in addition to MIDCAB vs. TECAB in its statistical analysis of robotic CABG techniques
		CPB time (min)	0 vs. 0 (p = NR)	
		AXC time (min)	0 vs. 0 (p = NR)	
		Complete revascularisation	71% vs. 72% (p = NS)	
		Reoperation for bleeding	8.5% vs. 3.7% (p = NS)	
		Reintervention	6.8% vs. 1.8% **(p < 0.005)**	
		MI	3.4% vs. 0 (p = NS)	
		Hospital length of stay (days)	5.5 vs. 6.5 **(p < 0.005)**	
		Mean follow-up (years)	1.8 vs. 2.5 **(p < 0.005)**	
		3-year reintervention-free survival	88% vs. 98% **(p < 0.005)**	

TECAB- Total Endoscopic Coronary Artery Bypass Grafting; MIDCAB- Minimally Invasive Direct Coronary Artery Bypass Grafting; PA-CABG- Port Access Coronary Artery Bypass Grafting; IMA- Internal Mammary Artery; CPB- Cardiopulmonary Bypass; AXC- Aortic Cross Clamp; RBC- Red Blood Cell; FFP- Fresh Frozen Plasma; PLT- Platelet; MI- Myocardial Infarction; PCI- Percutaneous Coronary Intervention; LAD- Left Anterior Descending Artery; RCA- Right Coronary Artery; MACCE- Major adverse cardiac and cerebrovascular events.

## 7 Discussion

CABG remains the gold standard treatment for complex multivessel coronary artery disease, resulting in superior long-term symptom relief, lower rates of reintervention, and improved survival when compared to medical therapy and PCI [4,[6], [7], [8]]. With the development of percutaneous revascularisation strategies, the demand for minimally invasive cardiac procedures has increased, with particular utility in isolated left anterior descending disease. Robotically assisted CABG has demonstrated comparable complication and long-term patency rates to conventional sternotomy, with additional benefits of decreased post-operative pain, improved cosmesis, reduced surgical site infection rate, reduced surgical trauma, decreased requirement for blood transfusions, shortened recovery time, and hospital length of stay [7,[9], [10], [11]]. Other studies reaffirm that there is no difference in peri-operative mortality and complication rates when compared to conventional CABG, however, greater patient satisfaction and quality of life scores are obtained with minimally invasive approaches [2]. However, controversy remains over the optimal robotic technique for coronary revascularisation with a variety of approaches quoted in the literature [7].

Kofler et. Al [2] in 2017 performed a prospective single-centre cohort study of patients with single vessel anterior wall disease between 2001 and 2014. Patients without contraindications to remote access perfusion and balloon aortic endoclamping underwent robotic TECAB with femoral-femoral cannulation and aortic endoclamping. Patients not suitable for robotic TECAB underwent robotically enhanced MIDCAB. Patients within the TECAB group had significantly longer operative times (292 min vs. 201 min, p < 0.001). Four patients in the MIDCAB group required conversion to CPB. There was a significantly higher conversion rate to sternotomy in the TECAB cohort (18% vs. 3%, p < 0.001). Transfusion rates of RBC (43% vs 6%, p < 0.001) and FFP (22% vs 3%, p < 0.001) were significantly higher in the TECAB group. Despite longer operative times, there was no significant difference in ICU/hospital length of stay (7 days vs 6 days, p = 0.716), post-operative stroke (1.5% vs 0, p = 0.454) or myocardial infarction (1.5% vs 0, p = 0.463). There were no peri-operative deaths in either group.

Similarly, Yang et. Al [3], performed a single centre prospective cohort study of patients with single vessel LAD or proximal RCA disease or multi-vessel disease in which the LAD was involved with non-LAD disease amenable to PCI. Patients with a localised lesion, total or subtotal occlusion of vessels were primarily selected for TECAB, whereas those with diffuse calcified disease, poor runoff, or myocardial bridging for scheduled for MIDCAB. Operative times were significantly shorter in the TECAB group, with no conversions to median sternotomy or peri-operative deaths. Post-operative coronary angiography or CT angiogram were utilised to confirm graft patency prior to discharge, with both groups obtaining 100%. There was no post-operative mortality, stroke or myocardial infarction in either group. 98.7% of patients had freedom from reintervention with similar requirement for revascularisation between the two groups (2% vs. 0.71%, p = NR), however, statistical analysis of outcomes was not performed, thereby limiting the utility of this finding.

Pasrija et. Al [4], completed a single centre retrospective study in 2018 comparing hospital cost and short-term outcomes with TECAB vs. robotically assisted MIDCAB. Importantly, the two cohorts were performed by different surgeons in different years, perhaps confounding their analysis. The TECAB surgeon had performed >200 cases and was considered experienced in this operative technique, whereas the MIDCAB surgeon had performed <75 cases and remained on the learning curve for the procedure. The study demonstrated that in experienced hands TECAB operating times were comparable to MIDCAB times (3.5hrs vs. 3.3hrs, p = NS). There were no significant differences between the two groups in terms of operative mortality (2% vs 0%, p = NS) or complication rates including reintervention (2% vs 2%, p = NS), prolonged ventilation (14% vs 12%, p = NS)and readmission (12% vs 14%, p = NS). Total hospital cost was significantly higher in the TECAB group (US $33769 vs. $22679, p < 0.001), largely due to the cost of robotic operating and stabilising equipment.

Jegaden et. Al [5], compared three techniques for minimally invasive coronary artery bypass grafting in 160 patients who required elective LAD revascularisation. There were two distinct surgical eras. Era 1: January 1998 to September 2003, where a Port-Access CABG (PA-CABG) or MIDCAB was offered, depending on suitability for peripheral femoral access cannulation and era 2: September 2003 onwards, where TECAB or robotically enhanced MIDCAB was offered. There were no conversions from off-pump to on-pump or to median sternotomy approach, however, 19 TECAB patients required conversion to MIDCAB procedure during the operation. Reasons included poor quality LAD, intra-myocardial LAD, pleural adhesions, stabiliser failure, limited anterior space and septal backflow. There were no significant differences among revascularisation completion (71% vs 72%, p = NS) or intervention time between the groups (3.4 vs 3.1, p = NS). The rate for reintervention was significantly higher in the TECAB group, with 6.8% requiring PCI intervention prior to discharge (6.8% vs 1.8%, p < 0.005). There was no significant difference in post-operative bleeding between the three groups (8.5% vs 3.7%, p = NS). At 3-year follow-up there was no difference in survival between the three groups, however, the TECAB group was significantly more likely to have had recurrence of angina or reintervention (freedom of intervention- 88% vs 98%, p < 0.005). The conclusions of this study are limited by the inclusion of PA-CABG within the statistical analysis comparing TECAB and MIDCAB.

## 8 Clinical bottom line

Studies comparing TECAB and MIDCAB directly are limited in both number and quality. Although two prospective studies have been performed, the median follow-up for all included studies is at best 3 years. All current studies are single centred, with different protocols for following up patients, different primary outcomes, and various levels of operating experience, suggesting significant heterogeneity. Moreover, there is also significant variation within the MIDCAB and TECAB techniques amongst the studies reported-including the experience of the surgeon, use of cardiopulmonary bypass, patient selection, and target vessel grafting strategies-highlighting the complexity of comparing these two minimally invasive procedures. Based on the available evidence, we conclude that TECAB is associated with a higher rate of transfusions, conversion to median sternotomy or MIDCAB, early graft failure and reintervention compared to the MIDCAB approach. We advise caution in adopting a TECAB approach.

## Ethical approval

Nil ethics approval required.

## Sources of funding

Nil funding for research.

## Author contribution

Lucy Manuel- Literature search, analysis and writing of manuscript.

Laura Fong- Analysis and editing of manuscript.

Hugh Wolfenden- Analysis and editing of manuscript.

Levi Bassin- Analysis and editing of manuscript.

## Registration of research studies

Name of the registry: NA-nil human participants

Unique Identifying number or registration ID:

Hyperlink to your specific registration (must be publicly accessible and will be checked):

## Guarantor

Levi Bassin.

## Declaration of competing interest

Nil conflict of interest to declare.
